# A Dual Load-Modulated Doherty Power Amplifier Design Method for Improving Power Back-Off Efficiency

**DOI:** 10.3390/s23146598

**Published:** 2023-07-22

**Authors:** Yi Jin, Zhijiang Dai, Xiongbo Ran, Changzhi Xu, Mingyu Li

**Affiliations:** 1 Xi’an Branch of China Academy of Space Technology, Xi’an 710199, China; 2 School of Microelectronics and Communication Engineering, Chongqing 400044, Chinamyli@cqu.edu.cn (M.L.)

**Keywords:** Doherty, power amplifier (PA), load modulation, efficiency enhancement

## Abstract

In this paper, the load modulation process of a Doherty power amplifier (DPA) is analyzed to address the issue of why designed DPAs have a very low efficiency in the back-off state in some cases. A general formula of the real load modulation process is also given for analyzing the load modulation of a peak PA matching network. This provides a new perspective for improving the back-off efficiency of a DPA. To improve the power back-off efficiency of a DPA, a dual load-modulated DPA (D-DPA) design method is proposed. The core principle of the proposed design method is to control the load modulation process from the carrier PA to the peaking PA based on the design method of the traditional two-way DPA. The efficiency of the peaking PA in the back-off region is enhanced, thereby improving the efficiency in the entire back-off region of the DPA. Based on the proposed design method, a D-DPA operating at 2 GHz is designed and fabricated. The test results show that the saturated output power and gain are 43.7 dBm and 9.7 dB, respectively, while the efficiency at 6 dB output power back-off is 59.2%. The designed D-DPA eliminates the efficiency pit of the traditional two-way DPA in the output power back-off region.

## 1. Introduction

Multiple-input multiple-output (MIMO) technologies are widely used in 5G mobile radio and applications in IoT. In recent years, they have also been widely used in sensors and wireless communication applications [1,2]. With the rapid development of wireless communication technology, amplitude modulation and high-order modulation modes, such as OFDM modulation, are often used in modern communication systems, which often result in the signal having a higher peak-to-average power ratio (PAPR) [3,4,5]. This inevitably requires the power amplifier (PA) to work in the output power back-off (OBO) region, and the traditional PA architecture usually exhibits low efficiency in the OBO region. This will lead directly to a serious reduction in the efficiency of the entire transmitter [6,7,8,9,10,11]. As a PA architecture with high OBO efficiency, the Doherty power amplifier (DPA) [12,13,14,15,16,17,18] has attracted the attention of a large number of researchers due to its simple circuit structure and easy broadband design.

After ensuring that a PA can be linearized [19,20], efficiency is a critical indicator [21,22]. As a result, improving the OBO efficiency of DPAs has become a research hotspot for researchers in recent years [22]. Ref. [23] provided the derivation of the efficiency expression for a two-way DPA in the presence of transistor nonlinear phase distortion. The OBO range and the OBO efficiency of the DPA were also improved through a complex impedance load. Ref. [24] solved the problem of reduction in the conduction angle of a class C peaking PA by controlling the active load modulation. Thereby, the OBO efficiency of the DPA under a large OBO range was improved. In [25,26], the influence of knee voltage on a DPA’s back-off performance was reduced through the rational design of load modulation impedance, and the OBO performance of the DPA was improved. Ref. [27] proposed an improved output synthesis network that mitigated the effect of active load modulation impedance mismatch on the performance of Doherty amplifiers and improved OBO efficiency. To sum up, researchers have conducted a lot of research to improve the OBO efficiency of DPA in recent years. However, the methods reported seldom pay attention to the active load modulation process of the peaking PA in the back-off region, which significantly affects the performance of a DPA, such as the OBO efficiency.

In the OBO region, the carrier PA of a traditional DPA reaches voltage saturation (the amplitude of the fundamental voltage is equal to the amplitude of the DC voltage) and maintains the highest efficiency of 78.5% through the load modulation process of peak PA so that a DPA can maintain high efficiency in the whole 6 dB OBO region. The load modulation mechanism in which the voltage saturation occurs only on the carrier PA is often called “single load modulation (S-LM)”. In S-LM, the peaking PA is just turned on and has a very low efficiency at the OBO point. This phenomenon causes the efficiency of the DPA to drop sharply around the 6 dB OBO point and then to increase slowly, finally reaching 78.5% with the increase in input power. The result is an obvious pit in the efficiency curve of the DPA with S-LM. In some cases, the efficiency deterioration can be very severe. This is because only two conditions should be satisfied in the traditional DPA design: (1) the peak PA only needs to be in a matching state at the saturated state; (2) when its matching network is connected to the main network, the main network cannot be short-circuited. However, according to the research described here, there may be severe efficiency degradation during the load modulation process following the traditional DPA design approach. Therefore, studying D-DPA would be very informative.

To solve this problem and to improve the power back-off efficiency of a DPA, a design method of dual load-modulated Doherty PA (D-DPA) is proposed in this paper, which enhances the efficiency of the DPA in the entire OBO region and eliminates the pit of the standard DPA efficiency curve. This implementation mechanism maximizes the real part of the equivalent impedance of the peak power amplifier in a low-power state, so that the voltage of the peak PA can be very large to increase the efficiency even when its output power is small.

This paper proposes a dual load-modulated Doherty PA design method for improving the OBO efficiency. In Section 2, we analyze the relationship between the network transmission phase and equivalent load, provide the derivation process of the load modulation process, and introduce a general design method of D-DPA. Then, a design example and the experimental results are given in Section 3. In Section 4, the conclusions are provided.

## 2. Analysis Of The Proposed D-DPA

### 2.1. Peak Path Load Modulation Process Under Fixed Phase Delay

**Figure 1:**
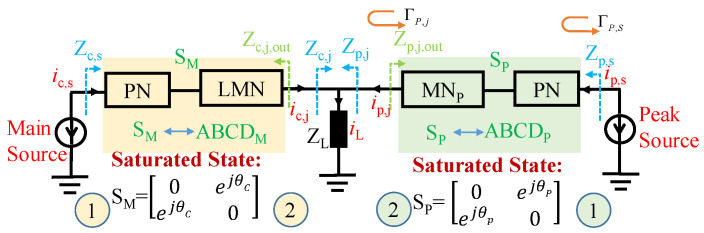
Schematic block diagram of the output matching network: $S_{M}$: main PA’s S parameter for the whole network containing the networks of PN and LMN; $S_{P}$: peak PA’s S parameter for the whole network; $\theta_{C}$: transmission phase of main PA; $\theta_{P}$: transmission phase of peak PA.

The output matching network (OMN) of a schematic block diagram of the proposed D-DPA is shown in Figure 1. For the S parameter networks $S_{M}$ and $S_{P}$, the reference impedances at the current plane and combination node are $R_{opt}$ and $2Z_{L}$, respectively, and $R_{opt}$ is the PA’s optimal impedance at the saturated state. The impedance $Z_{p,j}$ of the peak matching network at the combination point can be written as follows:

(1) $$\begin{array}{c} Z_{p,j}=\left(1+\frac{i_{c,j}}{i_{p,j}}\right)Z_{L}. \end{array}$$

Assuming that the peak matching network (MN) and the parasitic parameter network (PN) as a whole can create a peak transistor in a matching state in a saturated state, the cascaded S parameters of the two networks can be written as [17]:

(2) $$\begin{array}{c} \left[\begin{array}{cc} S_{11} & S_{12}\\ S_{21} & S_{22} \end{array}\right]=\left[\begin{array}{cc} 0 & e^{j\theta_{p}}\\ e^{j\theta_{p}} & 0 \end{array}\right]. \end{array}$$

If $max\left(i_{c,j}\right)=max\left(i_{p,j}\right)$, then the reflection coefficient at the combination node can be written as:

(3) $$\begin{array}{c} \Gamma_{P,j}=\frac{Z_{p,j}-2Z_{L}}{Z_{p,j}+2Z_{L}^{*}}. \end{array}$$

Then the reflection coefficient at the current plane can be written as:

(4) $$\begin{array}{c} \Gamma_{P,S}=\frac{S_{12}S_{21}}{1-S_{22}\Gamma_{P,j}}\Gamma_{P,j}+S_{11}=\Gamma_{P,j}e^{j2\theta_{p}}. \end{array}$$

And the input impedance of the peak PA at the current plane can be written as:

(5) $$\begin{array}{c} Z_{p,s}=\frac{Z_{opt}+Z_{opt}^{*}\Gamma_{P,S}}{1-\Gamma_{P,S}} \end{array}$$

where $Z_{opt}$ is the optimal impedance at the saturated state.

The efficiency of the peak power amplifier is calculated as follows

(6) ηpk=12Re[ZP,S]i1,pk21V0I0

where $V_{0}$ and $I_{0}$ represent the DC voltage and the DC current of the peak PA; $R_{opt}$ represents the real part of the optimal impedance at the saturated state; and $i_{1,pk}$ is the fundamental current of the peak PA at the current source. When the impedance modulation curve is on the left side of the Smith chart, it indicates that Re[Zp,s]<Ropt. Then, we can obtain

(7) ηpk=12Re[ZP,S]i1,pk21V0I0<12Ropti1,pk21V0I0.

Therefore, under this kind of impedance modulation trajectory, the efficiency of the peak PA is far less than that of the traditional class AB PA, which will lead to a severe efficiency “pit” in the DPA.

**Figure 2:**
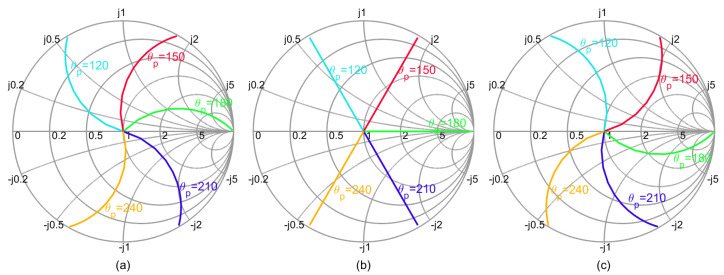
Load modulation process of peak PA vs. different conditions: (**a**) $Z_{L}=1+j$ (**b**) $Z_{L}=1$ (**c**) $Z_{L}=1-j$.

During the whole load modulation process, if the current at the combination point is in phase, the reflection coefficient of the peak PA at the current plane is shown in Figure 2. Different load impedances will bring different modulation trajectories. Therefore, it is necessary to study the corresponding relationship between the different loads and network phases to ensure that the load modulation trajectory meets the requirements of high efficiency. In the process of DPA design, it is necessary to appropriately select the load impedance and the phase of the matching network to make the load modulation trajectories approach the high efficiency state. Otherwise, the efficiency would be very low, such as the cure with $\theta_{p}=120^{\circ }$ in Figure 2a, the cure of $\theta_{p}=120^{\circ }$ and $\theta_{p}=240^{\circ }$ in Figure 2b, and the cure of $\theta_{p}=240^{\circ }$ in Figure 2c.

### 2.2. Phase Delay Variation Under Different Load

The parameters of the load modulation network can be characterized in the form of a transmission matrix, so the current relationship between the two nodes of the current source plane and combination node can be characterized as:

(8) $$\begin{array}{c} \left\{\begin{array}{c} v_{1}=Av_{2}+Bi_{2}\\ i_{1}=Cv_{2}+Di_{2} \end{array}\right.\to \frac{i_{2}}{i_{1}}=\frac{1}{CZ_{L}+D} \end{array}$$

**Figure 3:**
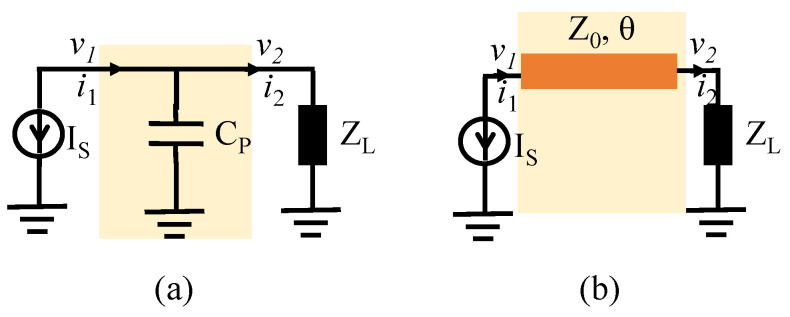
Phase delay analysis of two cases: (**a**) Parallel capacitor (**b**) Cascaded transmission line.

To further analyze the change in the network phase delay in the process of load modulation, we use a parallel capacitor circuit and classical transmission line network to study the phase variation between the current source and load impedance, as given in Figure 3. Their transmission matrix can be expressed as:

(9) $$\begin{array}{c} ABCD_{Cp}=\left[\begin{array}{cc} 1 & 0\\ \frac{1}{Z_{cap}}=j\omega C_{p} & 1 \end{array}\right] \end{array}$$

(10) $$\begin{array}{c} ABCD_{TL}=\left[\begin{array}{cc} \cos(\theta ) & jZ_{0}\sin\left(\theta \right)\\ j\frac{1}{Z_{0}}\sin\left(\theta \right) & \cos(\theta ) \end{array}\right]. \end{array}$$

**Figure 4:**
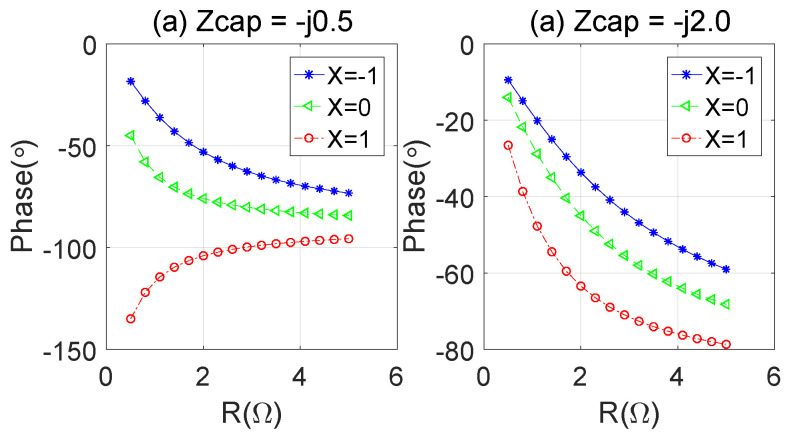
Phase delay vs. $Z_{L}$$\left(Z_{L}=R+jX\right)$: (**a**) Zcap = −j × 0.5; (**b**) Zcap = −j × 2.

In order to better observe the influence of different capacitance values on the network phase delay during load modulation, as shown in Figure 4, the variation in the phase delay, along with the load impedance, can be calculated. It can be seen from the figure that, under different loads of parallel capacitors, the phase delay characteristics of the network show other trends along with the load variation. In the high-power or high-frequency band, the parasitic parameters of the peak PA will greatly affect the load modulation effect. According to Formulas (4) and (5), different phase delays will bring different equivalent input impedances, so we need to pay attention to the peak load modulation.

**Figure 5:**
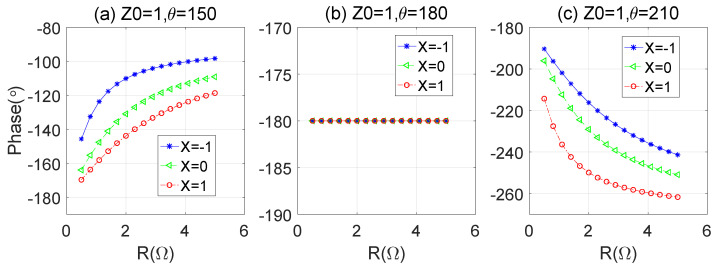
Phase delay vs. $Z_{L}$$\left(Z_{L}=R+jX\right)$: (**a**) $\theta =150^{\circ }$; (**b**) $\theta =180^{\circ }$; (**c**) $\theta =210^{\circ }$.

Similarly, given different transmission line electrical lengths and different load impedances, the phase delay value of the cascaded microstrip line can also be calculated according to the right part of (8); several groups of phase delay curves are shown in Figure 5. Observing the Figure, it can be seen that once the electrical length of the transmission line deviates from $180^{\circ }$, the phase delay is no longer a constant.

It can also be seen that the phase delay of the transmission line network will change during the load modulation process. This will enable the main PA to achieve a good matching effect even under low-power and high-power conditions, but the overall efficiency will be reduced due to the low efficiency of the modulation track of the peak PA. Therefore, the load modulation characteristics of the peak PA also need to be studied to avoid efficiency pits.

### 2.3. Real Dynamic Load Modulation Process

According to the previous analysis, it can be seen that the phase delay of the network changes with variation in the load impedance value. Therefore, during the load modulation process, the phase at the combination point is not fixed, and the equivalent impedance cannot be calculated simply according to the ratio of the output power. Instead, the modulation MN of the main PA and the MN of the peak PA need to be combined into a whole for analysis and design.

Assume that the relationship between the current of the main PA and the current of peak PA at the current plane has the following expression:

(11) $$\begin{array}{c} i_{p,s}=f\left(i_{c,s}\right) \end{array}$$

Then, $i_{c,j}$ and $i_{p,j}$ can be described as:

(12) $$\begin{array}{c} i_{c,j}=\frac{i_{c,s}}{C_{M}Z_{c,j}+D_{M}} \end{array}$$

(13) $$\begin{array}{c} i_{p,j}=\frac{i_{p,s}}{C_{P}Z_{p,j}+D_{P}} \end{array}$$

And we know that

(14) $$\begin{array}{c} Z_{c,j}=\left(1+\frac{i_{p,j}}{i_{c,j}}\right)Z_{L}\;\mathrm{and}\;Z_{p,j}=\left(1+\frac{i_{c,j}}{i_{p,j}}\right)Z_{L} \end{array}$$

Accroding to (12)–(14), we can obtain:

(15) $$\frac{i_{c,j}}{i_{p,j}}=\frac{i_{c,s}}{i_{p,s}}\frac{C_{P}\left(1+\frac{i_{c,j}}{i_{p,j}}\right)Z_{L}+D_{P}}{C_{M}\left(1+\frac{i_{p,j}}{i_{c,j}}\right)Z_{L}+D_{M}}$$

By rearranging the above formula, we can obtain

(16) $$\begin{array}{c} \alpha_{1}=\frac{\alpha_{0}\left(C_{P}Z_{L}+D_{P}\right)-C_{M}Z_{L}}{C_{M}Z_{L}+D_{M}-\alpha_{0}C_{P}Z_{L}} \end{array}$$

where $\alpha_{1}=i_{c,j}/i_{p,j}$ and $\alpha_{0}=i_{c,s}/i_{p,s}$.

Now, once the relation of $\alpha_{0}$ is confirmed, we can obtain the input impedance of the main and peak PA at the current plane.

(17) $$\begin{array}{c} Z_{c,s}=\frac{A_{M}\left(1+\frac{1}{\alpha_{1}}\right)Z_{L}+B_{M}}{C_{M}\left(1+\frac{1}{\alpha_{1}}\right)Z_{L}+D_{M}} \end{array}$$

(18) $$\begin{array}{c} Z_{p,s}=\frac{A_{P}\left(1+\alpha_{1}\right)Z_{L}+B_{P}}{C_{P}\left(1+\alpha_{1}\right)Z_{L}+D_{P}} \end{array}$$

The drain current of the power amplifier is mainly controlled by the gate voltage, so the current relationship between the main PA and peak PA can be adjusted through the power splitter and bias voltage at the input. To simplify the analysis, the drain current relationship described in (11) is rewritten as follows:

(19) $$\begin{array}{c} i_{p,s}=\left\{\begin{array}{cc} 0 & 0\leq i_{c,s}<0.5\\ 2i_{c,s}-1 & 0.5\leq i_{c,s}\leq 1 \end{array}\right. \end{array}$$

**Figure 6:**
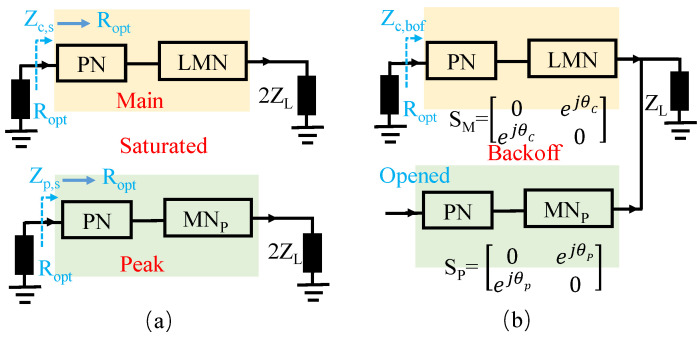
Design schematic: (**a**) Saturated state; (**b**) Back-off state.

The solution process of the D-DPA matching network is as follows: **(1)** As shown in Figure 6a, select the optimal impedance $R_{opt}$ and the load impedance $Z_{L}$, and design matching networks for the main PA and the peak PA, respectively, so that they are non-reflective at the source port in the saturation state. **(2)** Then, adjust $\theta_{C}$ and $\theta_{P}$ to put the main PA in a matching state in the back-off state. A corresponding schematic diagram is given in Figure 6b. **(3)** According to the above Formulas (12)–(19), the variation in the $Z_{c,s}$ and $Z_{p,s}$ at the current plane can be observed in the actual load modulation process based on Figure 1. In this way, the situations where the peak PA presents a low-efficiency state can be avoided.

To better explain the design process of the DPA some groups of obtained parameters are shown in Table 1. They all meet the conditions that the main and peak PA are in the matching state at the saturated state, and the main circuit also meets the matching state (continuous class B/J) in the back-off state. But the peak circuit has different characteristics in the load modulation process. The data are obtained by the following method: The transmission phase of the main circuit is first fixed, and then the transmission phase of the peak circuit and load impedance are obtained through optimization.

**Figure 7:**
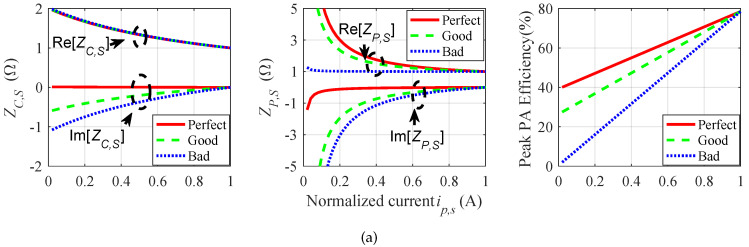

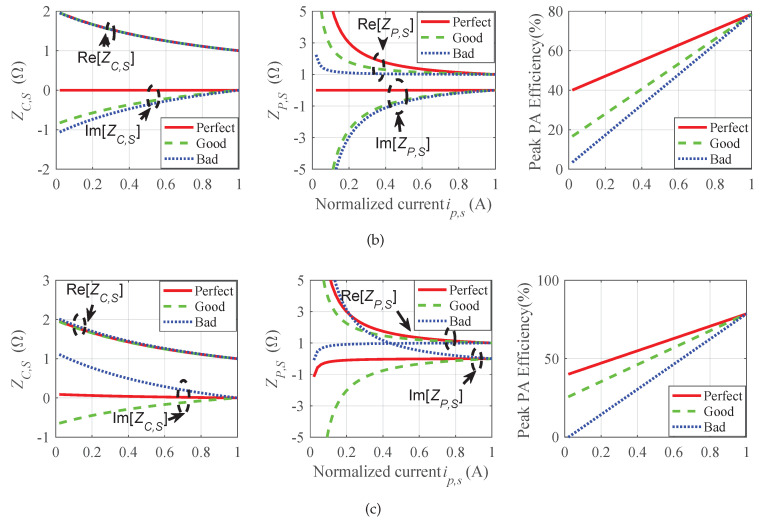
$Z_{C,S}$, $Z_{P,S}$ and peak PA efficiency during load modulation for the 3 cases: (**a**) $\theta_{M}=52^{\circ }$; (**b**) $\theta_{M}=90^{\circ }$; (**c**) $\theta_{M}=128^{\circ }$.

**Figure 8:**
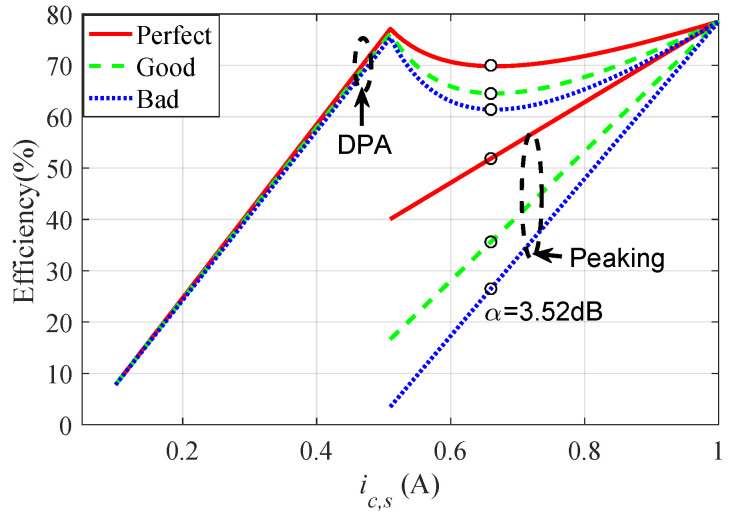
Efficiency during load modulation at $\theta_{M}=90^{\circ }$.

Correspondingly, Figure 7 shows a comparison diagram of three cases of the main power amplifier in different $\theta_{C}$. It can be seen from the figure that, although they all meet the design criteria of a DPA, the peak PA may have a low back-off efficiency in part of the solutions. Through efficiency conversion, the DPA efficiency under a poor load modulation will decrease by more than 8.6% compared with the optimal efficiency, as shown in Figure 8.

### 2.4. D-DPA Design Strategy

**Figure 9:**
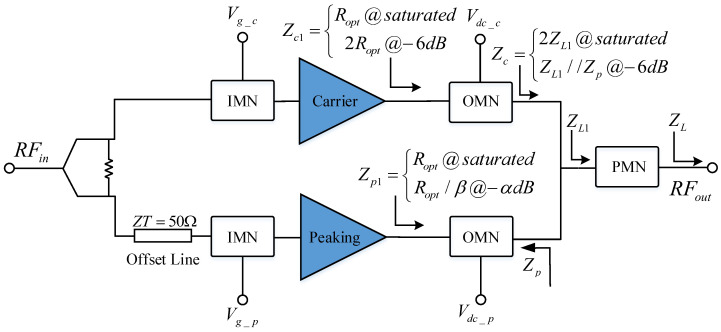
The structure of the proposed D-DPA.

To avoid the above-mentioned problem that the efficiency of a DPA decreases in the OBO region, a D-DPA design strategy is proposed in this paper, as shown in Figure 9, where α represents an OBO level between the 6 dB OBO point and the lowest point of the pit, 3.52≤α<6 dB, and β represents the normalized input voltage amplitude corresponding to the −α dB OBO point, satisfying $\alpha =-10\mathrm{lg}\beta^{2}$. The expression for the output power of the peaking PA at −α dB OBO point is:

(20) $$\begin{array}{c} P_{p}=\frac{1}{2}I_{\max}\left(\frac{V_{in}}{V_{\max}}-\frac{1}{2}\right)\frac{V_{in}}{V_{\max}}V_{DC} \end{array}$$

where $I_{\max}$ represents the maximum current allowed to flow through the peaking transistor.

The core idea of the proposed D-DPA design method is to add the load modulation process from the carrier PA to the peaking PA based on the design method of the traditional two-way DPA. This modulates the peaking PA to high efficiency at the −α dB OBO point. Therefore, the OBO efficiency of the entire DPA is improved compared with the traditional DPA with S-LM.

According to the design method of D-DPA proposed above, in order to satisfy the high efficiency of the carrier PA and the peaking PA in the saturation and OBO regions, their load impedances should meet the following expressions:

(21) $$\begin{array}{c} Z_{c,s}=\left\{\begin{array}{c} R_{opt}\;\;\;\;\;@\;saturated\\ 2R_{opt}\;@\;-6\;\mathrm{dB}\;OBO \end{array}\right. \end{array}$$

(22) $$\begin{array}{c} Z_{p,s}=\left\{\begin{array}{c} R_{opt}\;\;\;\;\;@\;saturated\\ R_{opt}/\beta \;@\;-\alpha \;\mathrm{dB}\;OBO \end{array}\right. \end{array}$$

However, the above Formula (22) is an ideal load modulation effect, which is difficult or even impossible to achieve in actual circuit design. We can only require $Z_{p,s}$ to be as high as possible during the load modulation process.

## 3. Design and Realization of the Proposed D-DPA

This section first provides the design process of the actual D-DPA and then verifies it through testing a designed D-DPA.

### 3.1. Design of the Proposed D-DPA

The core of the proposed D-DPA design method is to make the peaking PA highly efficient in the OBO region by controlling the load modulation process from the carrier PA to the peaking PA. Therefore, the design of the output network of the peaking PA is the focus of this design. Usually, the output network of the peaking PA only needs to meet the matching of the saturated point. But, in this design, the peaking PA needs to meet not only matching of the saturation point, but also matching of the OBO state, so that the peaking PA can achieve high efficiency in the OBO region.

According to Figure 8 and the efficiency calculation formula, the lowest point of the DPA efficiency curve in the OBO region is at α=3.52 dB. The efficiency curve decreases within the range of 3.52 dB <α< 6 dB, and the lower the input voltage is, the faster the decline rate is. Taking into account the lowest point of the efficiency curve, the decreasing rate of the efficiency curve, and the difficulty of output network design (proportional to the value of $R_{opt}$ in the OBO region), this design sets the modulation point of the peaking PA at α=4.5 dB. Commercial GaN HEMTs CGH40010F from Wolfspeed were used as the active devices for both the carrier and peaking PAs, with operation voltage $V_{DD}=28$ V, maximum current $I_{max}=2$ A, and maximum output power more than 40 dBm. Therefore, when the DPA was at the OBO point of −4.5 dB, the output power of the peaking PA could be calculated by (20): $P_{P}=32$ dBm. Therefore, the design of the output network of the peaking PA should not only satisfy the matching condition at the saturated state, but also the matching condition at the OBO point when its output power is 32 dBm.

The previous analysis process is conducted on the current source plane; the actual transistor includes a parasitic parameter network whose impact on the impedance transformation should be considered. One convenient method is to determine the optimal impedance through load-pull technology, which does not require obtaining parasitic parameter network parameters in advance. Only after satisfying the matching condition at the saturated state is the phase of the network adjusted to create a peak PA with high efficiency at one medium power in the OBO region.

**Figure 10:**
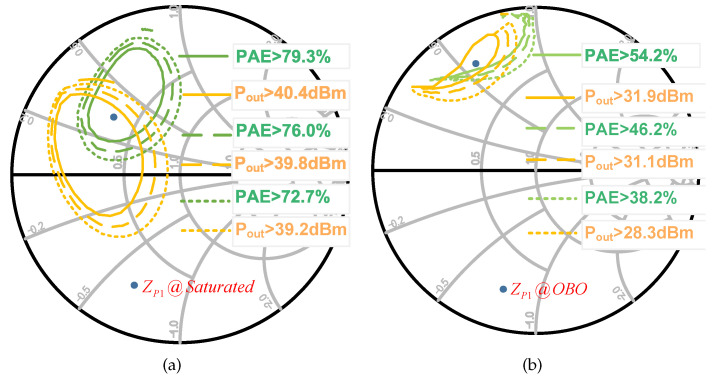
Matching results of the peak PA output matching at 2 GHz. (**a**) Saturated level; (**b**) OBO region.

An example is given as follows: As shown in Figure 10, the load-pull simulation results are presented in a Smith chart. First, the optimal impedance at the saturated state is given in Figure 10a. Under the trade-off between power and efficiency, the impedance $Z_{P1}$ at the circle marker is selected in the graph. Then, as can be seen from Figure 10b, the efficiency of the peaking PA in the OBO region reaches more than 54%, while the efficiency of the traditional method at the same level may be only 47%. That is, with the same output power, the efficiency of the different impedance positions varies greatly, which requires the selection of a high-efficiency impedance point. In this way, it can effectively avoid selecting low efficiency matching impedance, resulting in higher efficiency in the OBO region.

The design method for the matching network of the peak PA is given as follows: According to the previous load-pull simulation results, the network parameters are also optimized to ensure that the matching network of the peak PA meets the matching conditions in both the saturated state @ 40 dBm and the low power state @ 32 dBm. This ensures that the peak PA has high efficiency during the load modulation process.

**Figure 11:**
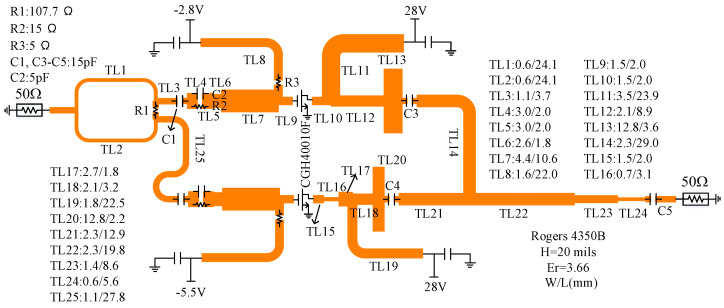
The PCB structure of the designed D-DPA.

When the peak circuit is determined, the matching network can be designed by using traditional DPA design methods. The output matching network also satisfies the matching of the saturated state (the peak PA is also saturated) and the OBO state (the peak PA does not turn on). The load impedance of the carrier PA $Z_{c}$ can be equivalent to $Z_{L1}//Z_{p}$ at the OBO level, where $Z_{p}$ is the output impedance of the peaking PA when it is not turned on; $Z_{L1}$ is the equivalent load impedance through the post-matching, and its value is 15 Ω. The PCB structure of the designed D-DPA is shown in Figure 11.

### 3.2. Realization and Experimental Verification

**Figure 12:**
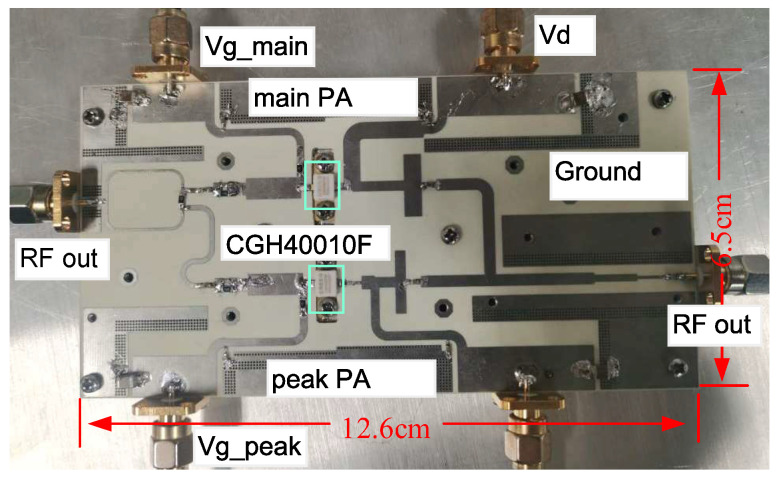
Photograph of the fabricated D-DPA.

A photograph of the fabricated D-DPA is shown in Figure 12 using a substrate of Rogers 4350B with 20 mils. In actual testing, the gate and drain of the transistor are powered through the SMA interface. The drain voltages of the peaking and carrier PAs are 28 V, and the gate voltages are −5.5 V and −2.8 V, respectively.

**Figure 13:**
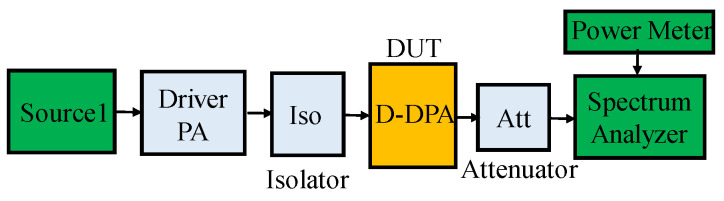
Testing principle block diagram.

The testing principle diagram is shown in Figure 13. The detailed testing process is as follows: first, measure the attenuation value of ATT, then measure the output power corresponding to ISO at different signal source powers, and record these data. Measure the output power of D-DPA under different signal source powers and record the corresponding DC current or DC power. By using this process, the gain, efficiency, and output power of D-DPA can be accurately measured.

**Figure 14:**
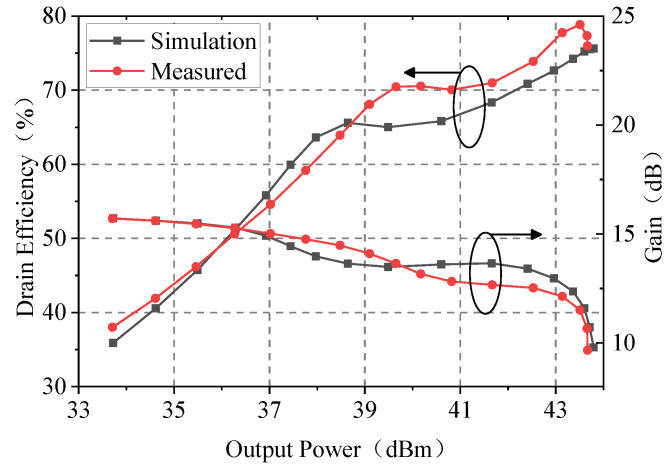
The simulated and measured results in terms of the drain efficiency and gain.

Under the experimental condition of continuous wave excitation, Figure 14 shows a comparison between the simulated and measured results in terms of the drain efficiency and gain at 2 GHz. Due to the error of the transistor model in class C mode and the difference between the electromagnetic simulation of the microstrip and the actual circuit performance, there is a certain gap between the test and simulation, but in general, these gaps are acceptable.

By analyzing the test results in Figure 14, it can be determined that the saturated output power and gain are 43.7 dBm and 9.7 dB, respectively. In contrast, the drain efficiency at the saturated point and the 6 dB OBO point are 76.0% and 59.2%, respectively. The test results are consistent with the simulation results. After the peaking PA is turned on, the drain efficiency curve of the designed D-DPA only shows a slight drop, but, compared with the large efficiency “pit” of the traditional DPA, this D-DPA shows a significant improvement.

Table 2 compares the D-DPA of this design and the 2-way DPAs reported in recent years with similar operating frequency and output power levels. From the table, it can be seen that the efficiency of our designed DPA in the back-off state is higher than that reported in most other literature. The performance of [28] is higher than ours because the harmonic impedance is simultaneously controlled, and the DPA operates in class F at the cost of sacrificing linearity. Our design did not intentionally control the harmonic impedance, so it is more similar to the techniques reported in the other articles. This also indicates that our design scheme has a good effect on improving the DPA back-off efficiency; that is, the D-DPA of this design has certain advantages in OBO efficiency and in other respects.

When designing and implementing an asymmetric DPA with 12 dB OBO, we found that efficiency pits would be become particularly severe, especially in broadband applications. Therefore, the defects in the load modulation process of the peak PA may be overcome by using the dual load modulation technique.

## 4. Conclusions

A D-DPA design method is proposed in this paper to improve the back-off efficiency of a DPA. The proposed design method of D-DPA enhances the efficiency in the OBO region by controlling the active load modulation of the carrier PA to the peaking PA, and greatly improves the efficiency pit in the OBO region compared with the traditional DPA design approach. Excellent OBO efficiency performance is achieved by the fabricated D-DPA through continuous wave experiments at the operation frequency of 2 GHz. Both theory and experiment confirm the effectiveness of this method. Further, this theory can also provide new ideas for improving the efficiency of the back-off region with a larger amount of OBO.

## Figures and Tables

**Table 1:** A group of solutions corresponding to Figure 1.

	$\theta_{C}$	$\theta_{P}$	$Z_{L}$	$Z_{c,\mathrm{bof}}$
Case 1: Perfect	52	144	1 − j0.8	2
	90	180	1	2
	128	222	1 + j0.8	2
Case 2: Good	52	154	1 − j1.6	2 − j0.6
	90	196	1 − j0.4	2 − j0.9
	128	230	1 + j0.3	2 − j0.6
Case 3: Not Good	52	163	1 − j2.2	2 − j1.1
	90	200	1 − j0.54	2 − j1.1
	128	196	1 + j2.2	2 + j1.1

Optimal impedance at saturated state is 1 Ω. $Z_{c,bof}$: impedance at back-off state; $\theta_{C}$: phase of $S_{M,21}$; $\theta_{P}$: phase of $S_{P,21}$.

**Table 2:** Compared with Reported 2-WAY DPAs.

Ref./Year	Frequency (GHz)	Pout (dBm)	DE@OBO (%)	DE@$P_{\max}$ (%)	Gain (dB)	Load Modulation for Peaking PA
[29]	2.2	43.6	54@9 dB	71	8.0	Not Given
[28]	2.4	44	74@6 dB	86.7	8.2	Not Given
[30]	2.15	43	55@6 dB	70	6.0	Not Given
[31]	2.1	41.8	50@6 dB	65	12.5	Not Given
This Work	2.0	43.7	59.2@6 dB	76.0	9.7	Yes

## Data Availability

The datasets generated during and/or analyzed during the current study are available from the corresponding author on reasonable request.
